# Editorial: Fighting microbial biofilms: novel therapeutics and antibiofilm strategies

**DOI:** 10.3389/fcimb.2025.1680391

**Published:** 2025-08-28

**Authors:** María Gabriela Paraje, Jordi Morató, Fernando Gomes de Souza

**Affiliations:** ^1^ Cátedra de Microbiología, Facultad de Ciencias Exactas, Físicas y Naturales, Universidad Nacional de Córdoba, Córdoba, Argentina; ^2^ Instituto Multidisciplinario de Biología Vegetal (IMBIV), Consejo Nacional de Investigaciones Científicas y Técnicas (CONICET), Córdoba, Argentina; ^3^ United Nations Educational, Scientific and Cultural Organization (UNESCO) Chair on Sustainability, Universitat Politècnica de Catalunya, Barcelona, Spain; ^4^ Federal University of Rio de Janeiro, Rio de Janeiro, Brazil; ^5^ College of Engineering and Computing, Florida International University, Miami, FL, United States

**Keywords:** microbial biofilms, innovations, associated infections, biofilm, antibiofilm, novel therapeutic agent

Biofilms present researchers with a range of challenges and opportunities, particularly due to their complex architecture, persistence and resistance to conventional treatments, and pivotal role in chronic infections (Almatroudi, 2024; Coenye et al., 2024; Paraje, 2018). In recent years, numerous antibiofilm compounds and alternative strategies have been identified, aimed at preventing biofilm formation or promoting biofilm dispersal to eradicate mature communities (Zhao et al., 2023). The articles compiled in this Research Topic address these pressing issues by focusing on the development of novel therapeutics, innovative techniques, and safe, effective alternative approaches, reflecting the rapidly evolving landscape of this critical field. The Research Topic also underscores the importance of interdisciplinary collaboration. By bridging diverse scientific disciplines—including microbiology, nanotechnology, pharmacology, and clinical medicine—these contributions offer a comprehensive and contemporary perspective on biofilm research, while proposing novel therapeutic avenues to tackle the global challenge of biofilm-associated infections (Paraje, 2023; Da Silva et al., 2024). Altogether, this Research Topic compiles state-of-the-art investigations into biofilm formation, maturation, and disruption, providing a forward-looking overview of both current challenges and future opportunities in the field.

This Research Topic opens with an insightful Mini Review by Grooters et al., who explore emerging interventions—including ultraviolet radiation, antimicrobial peptide design, phage therapy, and immunotherapy—as promising tools to combat and control pathogenic biofilms. The authors emphasize that contemporary medicine is engaged in a microbial arms race, a mounting global challenge that demands innovative strategies extending beyond conventional antibiotic therapies. Their review provides a compelling overview of alternative approaches that could reshape the future of biofilm management and antimicrobial resistance control. Despite years of effort to develop new antibiotics for eradicating multidrug-resistant and multi-virulent microbial infections, treatment failures and poor clinical outcomes remain common. Recognizing the pressing need for novel anti-virulence strategies, Bakeer et al. employed phenotypic assays, molecular docking, and genetic analyses to assess the anti-virulence potential of coumarin, simvastatin, and ibuprofen. These results highlight the promising role of ibuprofen as an adjuvant therapy, with the potential to enhance the efficacy of existing antibiotics against highly resistant and virulent *Staphylococcus aureus* infections, representing a valuable contribution to the ongoing search for alternative therapeutic approaches.

An additional innovative strategy for eradicating staphylococcal biofilms is presented by Grooters et al., who developed a localized drug delivery system using antibiotic-impregnated blood clots. Using murine blood clots to enhance antibiotic penetration in coagulase-negative staphylococci biofilms, their approach improved biofilm clearance in a preclinical model and highlights the need for further research into clot-biofilm interactions and their clinical potential in managing persistent infections. Furthermore, Gustafson et al. propose a novel therapeutic approach targeting a *Pseudomonas aeruginosa* biofilm-like structure using intrapleural enzyme-based therapy combined with antibiotic washes. This case demonstrates the clinical significance of biofilms in chronic and persistent infections and illustrates the potential of localized, enzyme-assisted interventions as valuable adjuncts to standard treatment protocols in complex post-surgical scenarios. The development of quorum-sensing (QS)-targeted therapies represents a promising adjunct to conventional antibiotic regimens in the fight against resistant bacterial pathogens. In this context, Pan et al. propose an innovative strategy to combat *P. aeruginosa* by targeting QS, a key regulator of virulence. Their approach combines QS inhibition with reduced antibiotic doses to control infection and limit resistance. Of seven synthesized cinnamoyl hydroxamates, two showed strong potential a**s** QS inhibitors, supporting the integration of anti-virulence therapies with existing antimicrobial protocols.

The potential of the novel anti-biofilm peptide CRAMP - 34 in targeting biofilms offers a compelling alternative to conventional disruption strategies, shifting the focus toward inducing biofilm disassembly from within. In their study, Liu et al. demonstrated that CRAMP - 34 enhances the motility of *Acinetobacter lwoffii* biofilm-associated cells, promoting biofilm dispersion and effective eradication. These findings position CRAMP - 34 as a promising next-generation biofilm-eradicating agent and highlight bacterial motility as a valuable therapeutic target in anti-biofilm research.

Natural compounds in combination therapies are increasingly recognized as promising tools to combat resistant biofilm-forming pathogens. In this context, Paramanya et al. investigated the effects of apocarotenoids, particularly crocetin, on *Staphylococcal* strains. The study suggests that crocetin holds strong potential as an adjunct to conventional antibiotic, as it helps reduce biofilm formation and enhances the effectiveness of standard treatments against clinically relevant pathogens.

A critical aspect of biofilm research is the accurate assessment of biofilm viability, which is essential for evaluating both biofilm formation and the efficacy of antibacterial treatments. Tchatchiashvili et al. explore an alternative staining method using calcein acetoxymethyl and TMA-DPH, a membrane probe that assesses residual biofilm biomass. This novel viability assay is presented as a promising and reliable alternative to traditional SYTO9/PI staining, providing enhanced consistency and sensitivity across various bacterial species.

In a significant step towards innovative biofilm treatment strategies, iminosugars have emerged as a distinctive class of compounds with promising biofilm-inhibitory properties. In this context, Kozień et al. employed an *in vivo* wound infection mouse model to evaluate the efficacy of PDIA, a synthetic iminosugar, in treating biofilm-associated skin infections caused by *S. aureus* and *P. aeruginosa*. The findings identify PDIA as a promising candidate for treating biofilm-associated skin infections, and underscore the therapeutic potential of iminosugars in chronic and drug-resistant infections.

Further emphasizing the growing importance of nanotechnology in antimicrobial research, the contribution by López Venditti et al. provides valuable insights into the antimicrobial and antibiofilm potential of biogenic zinc nanoparticles (ZnNPs). Through a comparative analysis, the authors demonstrate the effectiveness of these green-synthesized ZnNPs against both planktonic and biofilm-forming states of various pathogenic microorganisms. This study reinforces the application of nanomaterials as sustainable and multifunctional agents to combat biofilm-associated infections, a matter of particular importance in the context of global health.

Judan Cruz et al. explore *Aeromonas* isolates treated with a panel of phytochemicals, providing valuable insights into environmental microbiology and public health. Their findings highlight the promising potential of phytochemicals as antibiofilm agents in environmental biotechnology and advanced wastewater treatment technologies.

Finally, Mayorga-Ramos et al. provide a comprehensive review of bacteriophage-based strategies for biofilm control, highlighting the potential of phages and their proteins to disrupt established biofilms. Their review emphasizes the versatility of phages as targeted, adaptable tools for use in clinical, industrial, and environmental settings.

In conclusion, the twelve articles published in the Research Topic “Fighting Microbial Biofilms: Novel Therapeutics and Antibiofilm Strategies” underscore the dynamic and rapidly advancing landscape of biofilm research. Collectively, these contributions shed light on significant progress in our understanding of how biofilms can be effectively targeted and disrupted through innovative antibiofilm agents and therapeutic strategies that act upon critical stages of biofilm development.

The Topic Editors extend their sincere gratitude to all contributing authors for their high-quality work and to the reviewers and editors whose thoughtful feedback and scientific rigor greatly enhanced the content of this Research Topic. Their commitment and expertise were instrumental in bringing this Research Topic to fruition. We are equally thankful to our readers for their engagement and enthusiasm for this important and evolving field. It is our hope that this Research Topic will inspire interdisciplinary collaboration and innovation in the development of effective, targeted, and sustainable strategies to combat biofilms and their impact on human, animal, and environmental health, in alignment with the One Health approach.
